# The Evolution of China’s Foreign Talent Policy: the Case Study of Beijing

**DOI:** 10.1007/s41111-023-00239-7

**Published:** 2023-03-28

**Authors:** Lingyu Xu

**Affiliations:** 1 School of Sociology and Political Science, Anhui University, Jiulong Road 111, 230601 Hefei, China; 2 Brussels School of Governance, Vrije Universiteit Brussel, Pleinlaan 5, 1050 Brussels, Belgium; 3 Center for European Union Studies, Anhui University, Feixi Road 3, 230039 Hefei, China

**Keywords:** Foreign talent policy, International talent policy, Immigration policy, Zhongguancun Science Park, Permanent residence system

## Abstract

This paper aims to explore the evolution of China’s foreign talent policy (FTP) and the factors that contribute to those policy changes. Selecting Beijing as a case study, this paper explores Beijing’s FTP and divides it into three periods (1949–1978; 1978–2000; 2000 until now). It argues that at the point when the policy system becomes more institutionalized, more simplified measures are adopted. In particular, this paper applies a three-tier framework to detect the factors contributing to the policy changes as follows: (1) the deregulation of China’s scientific and educational systems, (2) emerging actors in the FTP institutional setting (Zhongguancun Science Park, private think tanks and social groups), and (3) market-oriented reform of the policy tools, e.g., China’s permanent residence system.

## Introduction

Since the late 1970s, especially since Deng Xiaoping’s famous talk on the introduction of talents on 8 July 1983, an increasing number of foreign talents and professionals have come to work in China. Their work has contributed to the country’s development. Since the first group of foreigners settled in China, the government has formulated and adopted many policies for managing and serving foreign talents. By now, FTP has become one of the most important parts of China’s immigration policy system.

However, foreign talents are not equally located across the country. Foreign professionals with various talents and foreign students are normally strongly attracted by specific policies and consequently have gathered in several international cities and areas. In view of local government autonomy, it is thus important to explore FTP at the regional level.

Beijing is chosen as a case study through which we will explore China’s FTP. In its new 2016–2035 development plan,^1^ the city has reaffirmed its strategic position as China’s political and cultural centre, spearheading international communications and scientific and technological innovation. For decades, it has performed these functions and by doing so, has continuously attracted international talents.

Studies of China’s foreign talent policies usually focus on foreign experts from the Soviet Union before the ‘Reform and Opening-up’ in 1978 (e.g., Shen 2009) and the foreigner’s situation in the new millennium (e.g., Huang 2013). There is also some institutional research into the introduction of talent (e.g., Gao 2012). Recently, Lu et al. (2022) have studied China’s International Talent Policy with a focus on student groups both coming in and going out of China. By examining how the talent policy changes, they aim to detect the driving forces behind the policy changes. Yang et al. (2022) also explore China’s strategic responses to international students facing the COVID-19 pandemic. In addition, there are studies on the internal or domestic migrant policies (e.g., Hung 2023) and the diversified public opinions on groups with different educational backgrounds (e.g., Ma et al. 2022). Yet, talent policies with a focus on foreigners are still less studied and explained through a theoretical lens.

**Fig. 1:**
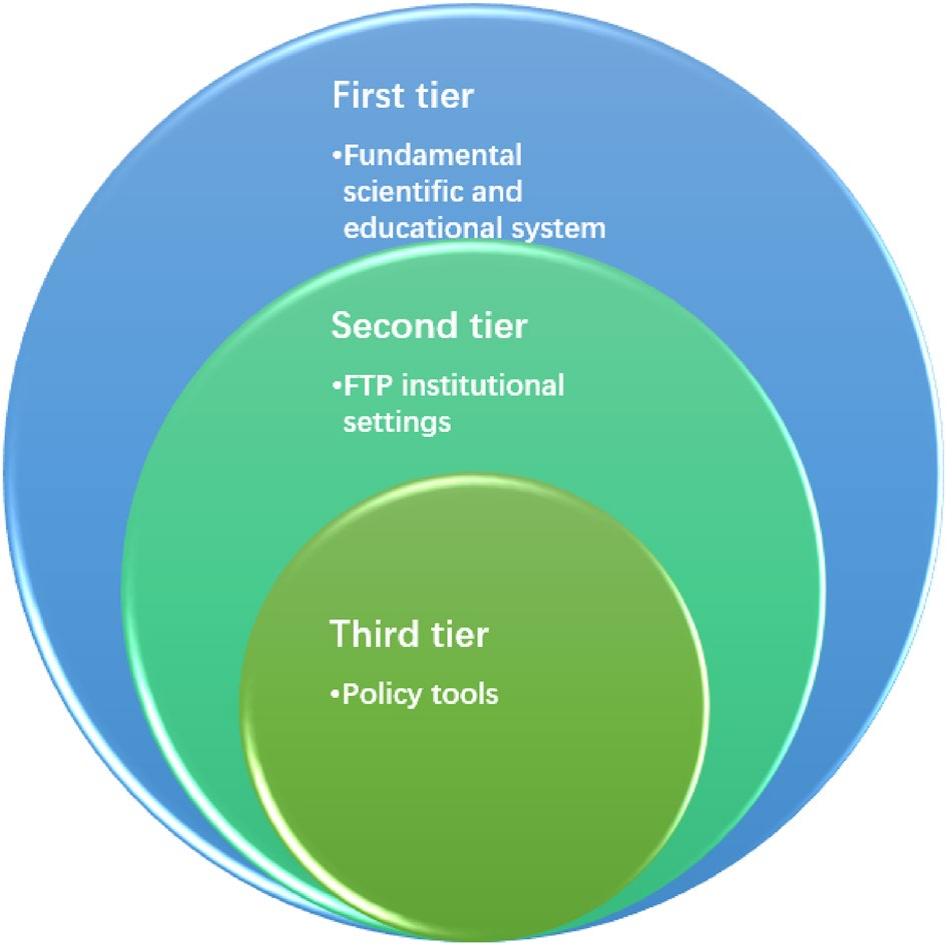
The three-tier theoretical framework constructed and applied in this paper

This paper aims to study the evolution of China’s FTP and the factors that contribute to those policy changes. With Beijing as a case study, the policy development will be divided into three periods, as presented in Sect. 2. We then aim to detect the policy-driving factors in Sects. 3 through 5. A theoretical framework reflecting a three-tier perspective is applied throughout the analysis (see Fig. 1); i.e., (1) examining the fundamental scientific and education system, (2) scrutinizing FTP institutional settings, and (3) examining the policy tools.

First, the reform of China’s scientific and education system is analysed in Sect. 3. It is argued that globalization provides the main impetus for China to change its fundamental scientific and education system as well as its FTP. The analysis that follows, in Sect. 4, is about the institutional settings of Beijing’s FTP. With the diversification of the actors involved in FTP decision-making, the migrant-related institutional settings have presented a movement from de-commodification to marketization. Third, Sect. 5 takes China’s Permanent Residence System as an example, examining the marketization methods in the evolution of China’s FTP. It demonstrates that not only the institutional setting, but also the FTP tools, have followed a market-oriented evolutionary trajectory. Overall, China’s reforms to its fundamental scientific and educational framework in response to globalization, the roles of the Zhongguancun Science Park and NGOs in the institutional settings of China’s FTP, and marketization as the main reform method of the permanent residence system, are key in the discussion. Section 6 presents the conclusion.

## Overview of Beijing’s FTP

Beijing’s FTP started from the establishment of the PRC in 1949. In the course of three main development periods, Beijing’s FTP system becomes well-constructed and institutionalized, especially after 2000. The FTP itself grows as a major part and can represent the dynamics of China’s immigration policy system. Furthermore, at the time when it becomes more institutionalized, fewer restrictions are observed.

### From 1949 to 1978: Initial Foreign Talents by Invitation Only

Foreign experts comprised the main foreigner group residing in China from the 1950s to the 1960s. Since the creation of the PRC in 1949, China has had a high demand for various experts and talents who could help to restore and develop the economy. At that time, China set its sights on the Soviet Union (Shen 2009). The initial introduction of experts was totally within the domain of diplomacy and negotiated by the senior officials of the two sides (Ibid., 2009).

After the issuance of the *Agreement on the Working Conditions of Soviet Experts in China* between China and the Soviet Union in 1950, China began to recruit a large number of Soviet experts (Ibid., 2009). The relevant ministries thus began to identify and assign key corresponding units to learn from the Soviet Union. These units needed to submit detailed requests for foreign experts to the central government, which would in turn provide a list of demands to the Soviet government.

Universities and colleges in Beijing normally took more responsibility for absorbing and promoting knowledge than universities and colleges in other places. The Beijing Medical College, for example, was the key unit appointed by the Ministry of Health at that time (Beijing Higher Education Chronicle Compilation Committee 2004: 329).

From the 1950s to the 1960s, Soviet experts, teachers and international students were the main foreigners residing in Beijing. However, their number was quite small. According to Table 1, until February 1960, of the 40 universities in Beijing, 13 universities contained 69 foreign experts, which included 35 Soviet experts, 10 experts from other socialist countries, and 37 experts from capitalist countries (Beijing Higher Education Chronicle Compilation Committee 2004: 330). 17 universities had introduced 64 foreign teachers, of whom 44 were from the Soviet Union, 2 from other socialist countries, and others from capitalist countries (Ibid., 2004: 330).

**Table 1:** Numbers of foreign experts and teachers by country categories in Beijing’s universities up to February 1960. Source: Beijing Higher Education Chronicle Compilation Committee. 2004. *Beijing Higher Education Chronicle (Vol. One).* Beijing: Huayi Press, p. 330

Country categories	Number of foreign experts	Number of foreign teachers
Soviet union	35	44
Other socialist countries	10	2
Capitalist countries	37	18

However, the FTP in the 1950s was totally within the domain of plan allocation, which gives it a feature of strong particularity. Foreign experts enjoyed a highly preferential policy whereby the Chinese government and the host units would ensure their entry, exit and stay in China. Introduction of Soviet experts was largely within the realm of special cases and needed special methods to handle it at that time. What is more, this period of introduction of Soviet talents soon ended, following the withdrawal of Soviet experts in 1960. In 1965, there were only 664 foreigners in Beijing (Beijing Local Chronicle Compilation Committee 2003: 398). In addition, the outbreak of the Cultural Revolution left almost no scope for the policy development. In 1977, following the end of the Cultural Revolution, only 180 foreigners remained in Beijing (Ibid., 2003: 398).

### From 1978 to 2000: the Start of the Institutionalized FTP

With the release of the nationwide *Regulation for Trial Implementation of the Work on Foreign Cultural and Educational Experts (1980),* China took its first step in legalizing its FTP. As a departmental regulation, this proposed regulation set out the principles, procedures, and hosting requirements involved in employing foreign experts in departments related to culture and education.

Foreigners with language ability formed the primary policy interest of the Beijing Government in this period. Some universities began to recruit foreign language teachers themselves, while some national ministries contributed to the introduction of foreign language teachers through their own channels. For example, the State Education Commission, together with the China Education Association for International Exchange, connected and cooperated with the English Language Institute, Educational Services Exchange with China, American Retired Teachers’ Organization, British Overseas Service Agency, etc., to bring more foreign language teachers into China (Beijing Higher Education Chronicle Compilation Committee 2004: 330). The Ministry of Metallurgical Industry chose to cooperate with Volunteers in Asia, US-CHINA Education and the Culture Exchange Centre (Ibid., 2004: 330).

From 1983, introducing foreign talents became *a ‘strategic principle of accelerating the construction of four modernizations in China’*, according to the *Decision on Introducing Foreign Intelligence for the Construction of Four Modernizations*. Soon afterwards, in September 1983, the State Council released the provisional rule on the detailed implementation of the decision.^2^ This provision—*Interim Provisions of the State Council on the Introduction of Foreign Talents—*removes limitations in cultural and educational fields. Foreign talents needed by key enterprises are listed. Furthermore, the provision gives more explicit instructions to the administrative departments in charge, and elaborates on the procedures for recruiting foreign experts. With the alleviation of the shortage of language teachers, Beijing shifted its policy focus onto other kinds of specialists.

At the end of 1983, there were 3921 long-stay foreigners in Beijing; this figure tripled to 12,076 at the end of 1987; in 1995, the number had increased to 29,025 (see Table 2). The number of foreign talents, including foreign experts, technicians and students also increased correspondingly.

**Table 2:** The number of long-stay foreigners in Beijing in 1983, 1987 and 1995. Source: author’s illustration, according to data from *Beijing Local Chronicle, Politics and Law Volume, Public Security Chronicle* (Beijing Local Chronicle Compilation Committee 2003)

	1983	1987	1995
Foreign experts and teachers	356	n/a	1114
Foreign engineers and technicians	146	n/a	
Foreign students	1445	n/a	7406
Others	1974	n/a	20,505
In total	3921	12,076	29,025

With these two legal texts, China’s FTP begins to be institutionalized. Foreign talents are administered and integrated into the regular legal framework. The high preference for invitation-only methods leaves the stage of history, showing a certain policy relaxation in the administration of foreign talents. Yet, national administration is still essential, and the strong characteristic of unified planning and administration remains. How the foreign experts should be treated by the hosting organizations is still regulated in detail.

### From 2000 Until Now: Active Talent Plans and the Simplified Foreign Talent Policies

#### A Series of Talent Introduction Plans

The FTP of the new millennium starts with various talent introduction programs whose distinctive features are high-level working conditions and superior treatment in their daily life provided for the introduced talents.

The ‘thousand talents plan’ was promoted by the Chinese central government at the end of 2008 with the aim of introducing high-level overseas talents. A parallel ‘ten thousand talents plan’^3^ was also announced in September 2012. Local governments then actively responded.

The Beijing government has issued a number of projects to join and support the central government’s plans. In 2008, the Beijing Government announced a talent project—the *Zhongguancun High-end Leading Talents Aggregation Project*.^4^ This program included the construction of world-leading scientific research centres, high-tech enterprise teams and entrepreneurial support teams. In the following year, the Beijing government introduced a special project for overseas talents—*Opinions on Implementing Beijing Overseas Talents Aggregation Project*,^5^ which aimed to establish Beijing as the most active and high-level talent capital for innovation and entrepreneurship in Asia. From 2014, the Beijing government carried out the *Beijing High-level Innovation and Entrepreneurship Talents Support Plan*,^6^ which was expected to coordinate with the ‘Beijing Overseas Talents Aggregation Project’ in constructing a High-level Innovation and Entrepreneurship Talent team-building system to enable these plans to link up with each other. In 2018, the Beijing government further promoted *Some Measures on Optimizing Talent Service, Promoting Scientific and Technological Innovation and Promoting the Development of High-end Industries,* whose important content dealt with strengthening support for the introduction of overseas talents. What is more, district governments promoted their own talent policies (see Appendix 1 for more information), which were all related not only to the introduction of domestic talents but also to that of overseas talents.

Besides the usual requirements for talents with particular cultural or educational backgrounds, these policies placed clear expectations and demands on candidates with an industrial background. For example, entrepreneurial candidates who wanted to apply under the ‘thousand talents plan’ were required to have overseas entrepreneurial experience or to have held middle and senior management positions in internationally well-known enterprises for more than 3 years, etc. Such changes indicated the Chinese government’s growing interest in supporting the country’s industrial development by seeking qualified personnel.

#### Supporting Policies

Talent introduction plans need supporting policies provided by different ministries and departments. These policy tools include visa policy, subsidy policy, housing policy, tax policy, medical policy and so on. Many of them have followed the path of preferential treatment since the last century. Yet, what is closely related to immigration regulations are the exit and entry policies which are mainly under the administration of the public security department.

Responding to those talent plans, the Ministry of Public Security has continued to make and upgrade the previous entry and exit regulations which affect the entry, exit and residence of the talents concerned. These ‘simplified’ policies mainly focus on the Zhongguancun Science Park, the ‘special talent zone’ in Beijing. Supporting policies include the ‘13 special policies’ in 2011, ‘8 measures’ in 2015, ‘20 measures’ in 2015, and ‘new 20 measures’ in 2018 (see Appendix 2 for more information). Many of these measures were implemented for the first time and included reduction in policy restrictions and simplification of applications. The permanent residence system and visa-related applications were the two most important policy breakthroughs.

First, the permanent residence system ushered in a breakthrough. Zhongguancun is implementing a points system which removes restrictions on certain types of work institutions and jobs for foreign applicants. The non-high-level talent foreigners (mainly foreigners from the new venture team and foreign technical personnel working in Zhongguancun Science Park) can thus benefit from this reform. Then, the procedures for applying for and renewing visas are also simplified. Foreign talents can renew their valid visiting visas many times. There are also fewer restrictions on international students who wish to find internships and conduct entrepreneurship activities following the reforms in Beijing and the Zhongguancun area. Foreign labourers can even get a private business residence permit if they are hired by foreign or Hong Kong and Macaw high-level talents, or innovative entrepreneurs with a permanent residence qualification or working residence permit.

At the end of 2018, the ‘thousand talents plan’ had introduced about 8000 overseas high-level talents in 14 batches to China.^7^ Meanwhile, 1486 talents introduced by that plan were based in Beijing at the end of 2016, 70% of whom were concentrated in the Haidian District.^8^ The number of high-level overseas talents in Beijing increased to over 1500 in 2018, accounting for about 20% of the total number in China. At the end of 2016, the ‘Beijing Overseas Talents Aggregation Project’ had cumulatively introduced 899 overseas high-end talents.^9^

There are also noticeable differences between the numbers of foreign experts in Beijing before and after the implementation of these talent plans. According to Table 3, the number of foreign experts working in Beijing increased from 26,696 to 32,982 in the years 2001 to 2007, showing an increase of 23.5% whereas this figure actually doubled from 32,982 to 78,093 in the years 2007 to 2013 when those talent plans were conducted.

**Table 3:** Number of foreign experts in Beijing between 2001 and 2013. What should be noticed here is that experts from Hong Kong and Macau were also calculated. Source: 1) State Administration of Foreign Experts Affairs, as cited in Huang 2013. *Research on foreigners in Beijing*. Beijing: China Book Press. 2) State Statistical Bureau and State Administration of Foreign Experts Affairs 2016. *2014–2015 compilation of statistical survey data from experts working in Chinese mainland*. China Statistics Press

Year	2001	2002	2003	2004	2005	2006	2007	2013
Number of foreign experts	26,696	19,598	28,118	28,332	32,402	21,802	32,982	78,093

Concerning the ‘permanent residence permit’, the ‘direct train’ policy in Zhongguancun Science Park was quite effective. Through this service, the procedures and approval time for applications for the permanent residence permit can be greatly simplified and shortened (to a maximum of 50 days).^10^ Just 3 months after the issuance of the ‘direct train’ policy in March 2016, the Zhongguancun committee had processed 91 ‘permanent residence permit’ recommendation letters for foreign high-level talents, 8 ‘residence permit’ recommendation letters for foreign talents, and 2 internal visa demonstration letters for international students in May 2016.^11^ By July 2018, a total of 468 permanent resident permits had been issued by the Ministry of Public Security through the ‘direct train’ channel in Zhongguancun Science Park. The National Immigration Administration in particular had issued 606 foreigner’s permanent residence ID cards in Beijing^12^ just 2 months after its official establishment in March 2018. However, it is not clear how many of those were issued to non-ethnic foreigners. According to a report forwarded by the Takongpao news,^13^ the first ‘batch’ of foreigners that got permanent residence through the ‘direct train’ in March 2016 were all ethnic Chinese. These foreign talents came mainly from companies located in Zhongguancun Park, e.g. Ying Huang (at the time vice president of the Lenovo Group), Minyi Chen (then senior director of Lenovo Beijing Co., Ltd.), and Bin Lin (then the President of the Xiaomi company) (Takungpao, 2016). So, it is unclear to what extent non-ethnic foreigners are involved in the foreign talent policies of Zhongguancun.

## The Deregulation of China’s Scientific and Education Systems

The globalization of science and technology, as one significant feature of globalization, has strongly induced countries to emphasize the introduction and exchange of talents and professionals. The period from the 1970s to the 1980s saw the beginning of a global multilateral scientific knowledge campaign (Ahmad 2014: 284). One important sign is the trend of a scientific renaissance in the newly industrialized countries of Asia and Latin America (Ibid., 2014: 284). This also stimulated China to actively end its relatively isolated situation. When Deng Xiaoping met Tanzanian vice president Mwinyi in 1985, he said *‘it is impossible for any country to develop and isolate itself. Without strengthening international exchanges and introducing advanced experience, science and technology and capital from developed countries, it is impossible’*.^14^ Recognition of the importance of international exchange demonstrated the willingness of the Communist Party of China (CPC) and the Chinese government to integrate into the international market. So far, the deep connection and participation in globalization is still considered to be beneficial to China’s national interest (Boylan et al. 2021: 36). How they reformed the domestic policy system to coordinate with international interactions would provide the key to their next actions.

As early as 1981, the National Science and Technology Commission had reported initial guidelines for the development of China’s science and technology.^15^ Four years later, in March and May of 1985, the Central Committee of the Communist Party of China (CCCPC) released formal decisions to reform China’s science and technology system as well as its educational system.^16^ These several important documents set the principles of reforms which involve (1) marketization reform, (2) deregulation of institutions and personnel, and (3) international interactions.

First, marketization became a priority during the reform. The decision to reform China’s science and technology system directly pointed out: ‘*At the same time of carrying out planning management for key national projects, we should use economic leverage and market regulation to make scientific and technological institutions have the ability of self-development and the vitality to automatically serve economic construction*’. This indicates that besides the plan allocation, market allocation is encouraged as a means of reforming the scientific and educational system. In addition, the coordination of science and economics is repeatedly emphasized in the central government’s conferences. Actually, promoting economic development was even clearly proposed as the primary task for the development of science and technology by the National Science and Technology Commission.

University–industry cooperation was then adopted in the reform. Besides state-owned companies, private science and technology enterprises were also encouraged in improving the combination of scientific research and production. In addition, the industrial development sphere was supported by the Chinese government with special promotional policies in several intellectual resource-intensive areas (CCCPC 1985a). Such reforms helped to bring about the establishment of the New Technology Industry Development Zone in Zhongguancun in 1988.

Secondly, the deregulation of universities and researchers constituted further significant content. The CCCPC admitted that ‘*(we) should expand the autonomy of higher education institutions and strengthen their links with production, scientific research and other aspects of society so as to enable them to actively adapt to the needs of economic and social development’* (CCCPC 1985b). By *‘creating a good environment for talents to emerge and people to do their best’,* individuals were allowed to establish scientific or technological service institutions and could get support and help from central or local governments (CCCPC 1985a).

In line with the ‘Reform and Opening-Up’ policy starting in 1978, focusing on international interaction was the third important part of the reform. Improvement in science and technology could not take place without personnel exchange. As reflections of the two decisions, not only were Chinese scholars and scientists able to conduct international activities abroad, but in addition foreign researchers were welcome to do research in China.

Zhongguancun Science Park is a particular case of China’s effort to participate in the globalization of science and technology. Its construction began in the early 1980s and it became China’s first National Hi-tech Industrial Development Zone in 1988. After more than 30 years of development, Zhongguancun Science Park has become a ‘special talent zone’. Reflecting these technological achievements, China is now regarded as being on the road to technological advancement (Mahoney 2023). And it is even argued to show a ‘digital orientalism’ trend, starting around 2017 (Ibid., 2023: 14), which enables China to tap its ‘global data power’ (Huang and Mayer 2023: 25).

These reforms show that in the face of globalization, especially the globalization of science and technology, China has started to reform its fundamental scientific and educational systems so as to be more competitive. More flexible industrial policies and deregulation of research institutions and researchers have been introduced since the 1980s. The reforms to the policy arena in the science and education systems thus provide a systematic preparation for institutional changes in the FTP.

## The Diversification of the Institutional Actors in the FTP

Institutional changes in the FTP are also in line with reforms in the decision-making process since the 1970s. China embarked on a reform aimed at realizing the ‘democratization’ and ‘scientificalization’ of decision-making^17^ to reflect criticisms of the high concentration of decision-making power, plus the non-institutionalized decision-making process as regards China’s political system before 1978 (Zhou 2011: 109; Shen 2013). The policy outcomes are thus inevitably influenced by both the formal institutional actors and informal institutional actors (e.g., Zuo 2022; Song 2022). With the reform, more actors have become involved in FTP’s decision-making. Zhongguancun Science Park has been set up and becomes the policy pioneer. Besides this, the role of NGOs is growing.

### ‘Early and Pilot Implementation’ and Zhongguancun Science Park

From the beginning of the reform, China has chosen a gradual implementation. The basic idea is to select specific areas to take the lead in experimentation and innovation within a limited range, to sum up the lessons from its successes and failures, and then to popularize the successful experience (Liu 2015). This reform model is usually called as ‘early and pilot implementation’ policy in the Chinese government’s normative documents (Ibid., 2015). Even though such practice is also regarded as policy adaptation by the local governments (Tsai and Tian 2022: 676), it reflects the empowerment of local authorities by the central authority.

The Chinese government also follows the principle of ‘early and pilot implementation’ in the field of China’s immigration policy. For the FTP, there is a general framework at the national level, while the central government allows local governments to make innovations and promote their own plans based on their specific requirements. It is thus not difficult to understand how, at the time when the CCCPC promoted the ‘thousand talents plan’ in 2008, the other central departments, provincial authorities, and district-level governments made their own talents plans. These plans are normally not uncorrelated, but are interconnected, the contents even overlapping with each other.

This ‘first try’ feature is apparently reflected in the development of Zhongguancun Science Park in terms of institutional changes in the FTP. The expectation in constructing this science park has remained that of leading the high-tech and innovation industry in China. From 1988, it evolved from the national high-tech industrial development zone to become, in 2009, the national independent innovation demonstration zone (see Appendix 3 for more information), while the past decade has viewed Zhongguancun Science Park as the policy pioneer of FTP dynamics. The central government and Beijing government released successive documents^18^ aimed at constructing Zhongguancun Science Park as a ‘special talent zone’ in 2010. According to the regulation, its special position allows it to operate *‘special policies, special institutions’* and to ‘*handle special cases with special methods and take the lead in establishing the talent priority development strategy in the overall situation of economic and social development*’.

Even though the Zhongguancun committee has high autonomy in making its own talent policy, trans-department cooperation has become essential in producing the FTP. For the years 2010 to 2014, 17 government departments announced 13 supporting measures which included policies on exit & entry, residence, integration and employment, yet these policies were quite general and did not provide enough guidance for the implementation process. Focusing on the policy implementation, more measures have been released since 2015. The Ministry of Public Security announced 20 new measures, 10 of which specifically applied to Zhongguancun Science Park and were implemented for the first time in China. In addition, in 2018, a further 20 measures were put forward by the Beijing Municipal Committee and Municipal Government, the Central Organization Department and the Ministry of Science and Technology, etc. As a policy outcome, the FTP is not only limited to the small groups in those projects, but is also applicable to young international students, foreign members of entrepreneurship teams, and even the families of these high-level talents in Zhongguancun. In recent years, there has also been a trend towards similar policies spilling over to other cities, or even on the national scale.

The aim of combining Zhongguancun’s function as the hi-tech industrial zone and talent zone is self-consistency in response to the globalization of science and technology, as the industry accumulation demands a flexible talent policy to assist in its rapid development. Zhongguancun Science Park thus reflects the institutional changes causing China to reform its FTP. It becomes the key actor in contributing policy changes and works within a trans-cooperation institutional context.

### The Growing Role of NGOs in the Decision-Making Process

#### Think Tanks

Think tanks in China are mainly state-funded and directly serve certain government ministries or the CPC (Morrison 2012; Tsai and Tian 2022). Beyond these government-funded ones, additional think tanks with private funding have been gradually developing since the 1990s, especially following Deng Xiaoping's tour to the South in 1992 (Wang et al. 2016: 897). This kind of think tank is normally called a ‘folk think tank’ or ‘private think tank’ to differentiate it from state-funded think tanks. The Chinese government and central committee of the party began to expand their advisory network to ‘folk think tanks’ to get rid of total dependency on those ‘internal think tanks’ from the 1990s (Wang and Fan 2013: 103). Independent decision-making advisory bodies therefore have gradually come to play an important role in the evolution of the reform of China’s decision-making system by engaging in a diversified competition with its counterparts under certain conditions (Zhou 2011: 113; Wuthnow and Chen 2021: 382).

The role of think tanks is also paid attention to by senior officials in China (Wuthnow and Chen 2021: 381). China’s current president Xi Jinping, for example, has repeatedly issued important instructions on the construction of think tanks in China in recent years, which is interpreted as part of the ‘new think tanks’ campaign (Ibid., 2021: 381). Besides, developing the ‘think tank’ was put forward for the first time in a CCCPC document—*Decision of the CCCPC on Several Major Issues Concerning the Comprehensive Deepening of Reform*^19^ in 2013.

Private think tanks are emerging within China’s migration policy-making process. The Center for China & Globalization (CCG), for example, is a private think tank with an emphasis on the study of the talents competition system and the migration system. It is registered under the United Front Work Department of the CCCPC. The structure of the CCG has two prominent features. First, its funding is mostly from companies; most of the senior vice president members and other vice president members come from large enterprises (CCG 2017: 85). Second, most of the founders and members of the CCG council have worked or are somehow linked with the Chinese government and international organizations (Ibid., 2017: 85). These two features show that the link between the private think tank and the government is still close, although the CCG is not administered by the government or the party directly. In recent years, CCG has demonstrated its influence through various works. On one hand, it undertakes dozens of talent policy related projects (including research into the points system and exit & entry policy for Zhongguancun Science Park) directly for government ministries and some local governments. On the other hand, the CCG also actively manages to submit advice and suggestions to the CCCPC and related government authorities. In 2016, leaders of the National Development and Reform Commission also visited the CCG to consult research concerning the establishment of the National Immigration Administration. Yet, it is acknowledged that, even though some talent policies which were released by the government were somehow related to the think tank’s advice, the degree of its influence is unclear.

#### Other Groups

Recent developments in Zhongguancun also show the increasing influence of NGOs. In 2016, ‘Zhongguancun the Belt and Road Industrial Promotion Association’ was initiated by mainly high-tech enterprises, relevant institutions and individuals to serve the development of innovative industry.^20^ In the area of international talents, its Cirrus Program has involved more than 200 companies and some universities in facilitating cooperation between international talents and Chinese enterprises. As Zhongguancun the Belt and Road Industrial Promotion Association has also been supported by the National Development and Reform Commission, the Ministry of Science and Technology, and relevant departments of Beijing, it can directly give feedback or suggestions especially to the Ministry of Science and Technology, or the Zhongguancun Science Park committee. Therefore, it is possible for Zhongguancun the Belt and Road Industrial Promotion Association to exploit support from the government as well as resources from hi-tech companies to function as a commercial platform linking the national strategy, high-tech enterprises, and international talents.

Based on the fact that the funding bodies of both the private think tank and Zhongguancun the Belt and Road Industrial Promotion Association are mainly large companies, the interactive activities of these two kinds of NGOs with the Chinese government can be regarded to some extent as forms of lobbying for certain interest groups. However, considering their close connection to the Chinese government, these NGOs actually play more of a complementary role in the decision-making process affecting FTP in China.

In sum, the FTP system is experiencing institutional change featuring diversified actors in the decision-making process. First, transforming the existing hi-tech zone—Zhongguancun Science Park—into the talent zone directly grants Zhongguancun high-level autonomy in creating its own talent policies. With policy support from various departments, plus trans-department cooperation, the dynamic status of Zhongguancun Science Park, as part of the Beijing government, indicates the adjustment of the institutional setting towards greater marketization in terms of the FTP. The increase in groups of talent benefitting from the Zhongguancun talent policies reflects China’s strategic change towards obtaining more human resources. Furthermore, as those changes in Zhongguancun Science Park are always related to industrial development, a clearly marketized change is evident in the migrant-related institutional setting. Secondly, the emergence of NGOs becomes another new phenomenon. Their links with large companies can add the weight of assured industrial benefit to China’s immigration decision-making process. Yet, their role in migration policy change is mainly complementary. The evidence from Zhongguancun and some NGOs has shown that the institutional setting of the FTP has changed from de-commodification towards more marketization on a significant scale (mainly within the Zhongguancun Science Park), with the potential to spill over to bigger areas.

## The Marketization of the Policy Tools for the FTP

The permanent residence policy carries weight in China’s FTP, as it is a valuable policy tool adopted by the Chinese government throughout its path of attracting and keeping foreign talents. In fact, the consideration of attracting and integrating foreign talents runs throughout the evolution of the permanent residence system. Initiation of the permanent residence measure was explicated as a measure to ‘*create a good environment for innovation and entrepreneurship for overseas talents in China’ in a notice jointly released by more than 20 departments in 2012.* Starting from an unstructured status, the permanent residence system has gone through a structured but rigid administration from 2004 to 2014, and a market-oriented reform from 2015 until now.

### Before 2004: The Unstructured Administration of the Permanent Residence System

Permanent residence status, as a kind of long-term residence, mostly represented an honour for foreigners living in China, especially before 2004. The grant of a permanent residence permit mattered not only to the foreign applicants, but also to the Chinese government, since such an honour was accorded to foreigners who had made important contributions to China. The Chinese government might organize a celebration ceremony for selectees to show the importance of the event. One example can be found in the ceremony organized by the Hunan Public Security Bureau in 2001^21^ (Rednet, 2001).

However, such symbolic meaning of the permanent residence system left almost no space for ordinary foreigners. Except for the first year after the release of the *Law of the People's Republic of China on Control of the Entry and Exit of Aliens* in 1986, when 24 foreign experts were granted permanent residence status in China (Beijing Local Chronicle Compilation Committee 2003: 399), only about 70 ‘international friends’ obtained permanent residence status during the following 15 years up to 2004. Another 3000 people who gained residence status were normally ethnic Chinese.^22^

### From 2004 to 2014: The Rigid Administration of the Permanent Residence System

The lack of regulations on permanent residence in China ended in 2004 when the *Measures for the Administration of Examination and Approval of Foreigners' Permanent Residence in China* were released. China’s permanent residence system is thus built on this measure. It classified permanent residence into four categories: investment, technology, family reunion and special contributions. For each category, it lays down relatively clear requirements. However, except for the family reunion card, the other kinds have quite high requirements in terms of company position, investment amount, academic position or contribution.

This policy priority assigned to foreign talents is also reflected in the data. Until March 2015, there were in total no more than 5000 foreigners^23^ who obtained the permanent residence card in China, while 1306 foreigners were processed through the high-level talents introduction plans, such as the ‘thousand talents plan’, in the period up to the 23rd of May, 2014.^24^ This reflects the fact that the thresholds for applying for permanent residence cards were still too high for ordinary foreigners to meet. Even the Overseas Chinese Affairs Office of the State Council released an article on its website stating that China’s ‘green card’^25^ was the most difficult card in the world to obtain.^26^

### From 2015 Until Now: The Marketization of the Permanent Residence System

The permanent residence system has undergone market-oriented reform in recent years. The Ministry of Public Security issued several exit & entry documents in response to Beijing’s talent work since 2015. Introducing the points system into the permanent residence system became the most distinctive reform.

Based on the *20 Exit & Entry Policies of the Ministry of Public Security in Support of Beijing's Innovative Development* (or the ‘20 measures’), the Beijing Municipality Public Security Bureau and relevant departments were authorized to formulate the points system. Zhongguancun Science Park became the first district to operate it in 2016.

The points system sets relatively clear standards for foreign applicants. In practice, it lowers the traditional thresholds and increases the applicants’ chance of obtaining the permit. Hong Guo, the previous deputy secretary and director of the Party Group of the Zhongguancun Administrative Committee, made this more explicit, and agreed that the points system policy was ‘*considering foreigners who cannot meet the criteria for “direct train” service but make positive contributions to Zhongguancun […] the points system is applicable to the foreign members of new venture teams in Zhongguancun and the foreign technical talents employed by Zhongguancun enterprises’.*^27^

Another policy, the ‘new 10 measures’ expanded the points system for applicants from those in the science and technology field to those in the service industry, which includes the finance, science, culture, commerce, tourism, etc. industries. It was first applied in the Chaoyang and Shunyi Districts in 2017. In fact, it was acknowledged as the expansion of the ‘20 measures’ to service industries in a range of pilot demonstration zones (Overseas Chinese Affairs Office of the State Council, 2017).

In sum, the evolution of China’s permanent residence system follows a market-oriented path. Before the release of the specific regulation on foreigners’ permanent residence qualifications in China, there was no standardized procedure which could guide foreigners in applying for them. Granting permanent residence qualification to foreigners was normally regarded as an honour conferred on foreigners who had made distinct contributions in China. So, the permanent residence qualification depended much on the plan’s allocation. Moreover, although the *Measures for the Administration of Examination and Approval of Foreigners' Permanent Residence in China* were released in 2004, the threshold for applying for permanent residence was still too high for most foreign talents to meet. The reforms in Zhongguancun Science Park introduced more market-oriented practices and measures into the permanent residence system from 2015. Besides high-level foreign talents in the science and technology field, more and more foreigners who work in the new venture teams or are hired by Zhongguancun companies can now gain the permanent residence card through a points system. In 2017, the Beijing government even expanded the reform to the service industry in another two districts of Beijing, bringing the reforms to a deeper and broader expansion, not only in the sense of geography, but also in the sense of industry. The reforms of the permanent residence system thus show China’s pursuit of increased competitiveness and represent the marketization of the policy tools for China’s FTP.

## Conclusion

China’s FTP, represented by that of Beijing, has mainly experienced three periods of development. First, in the initial period following the establishment of China in 1949, foreign talents or foreign experts were the main foreigner group living in China. Policies concerning these foreign talents directly laid the foundation for China’s immigration policy. However, foreign experts during this time were in China by invitation only. Foreign talent policies were highly preferential towards foreign experts, which made them lose generality. This situation changed at the end of the 1970s, when the Chinese government began to resume its communication with personnel in other parts of the world. Formal regulations concerning foreign talents were set, which established the institutionalization path for China’s FTP. The third period featured various talent introduction plans and is marked by fewer restrictions and simplified exit & entry policies.

The factors in the FTP policy changes can be traced in the light of a three-tier framework consisting of ‘system–institutional setting–policy tools’. At the system level, driven by the trend towards globalization of science and technology in the 1980s, China’s decision-makers decided to conduct a fundamental reform of its rigid scientific and educational system. Deregulation happened, moving from industry to education and to the research arena. With the rising autonomy of those institutions, the development of international interactions was to follow, and foreign talents became welcome in China again. System reforms thus gave fundamental and systematic support to the subsequent changes in institutional settings of the FTP.

The institutional setting of the FTP has become more diversified in recent years, as additional actors are brought into the decision-making process. The Zhongguancun Science Park in particular becomes the pilot zone for the FTP, which means either that the upper-level governments tend to apply the newest policies here, or that the Park itself has the autonomy to adopt its own talent policies. Considering Zhongguancun’s status as the innovative industry zone over many years, the practice of listing it as a ‘special talent zone’ clearly conveys the intention of promoting Zhongguancun’s competitiveness by introducing (foreign) talents. Other NGOs, such as private think tanks, also emerge into the decision-making process. Their involvement gives the FTP institutional setting more flexibility. The diversification of the institutional actors involved thus brings the sense of marketization to the FTP’s institutional setting, while the de-commodification feature is fading away along the path of policy evolution.

The third factor focuses on the policy tools (represented by the permanent residence policies) of the FTP. During the development of the permanent residence system from an unstructured situation to a rigid administrative and marketized reform, its threshold has been lowered and become more accessible to foreign groups living and working in Zhongguancun Science Park. The points-based reform is even overflowing to other districts of Beijing, which makes the adoption of the permanent residence reform on a larger scale more likely.

## Data Availability

The author confirms that the data generated or analysed during this study are included in this published article [and/or] its supplementary materials.

## Appendices

### Appendix 1: Talent projects conducted by Beijing district governments from 2009 to 2017

**Table:**

Beijing district governments	Talent projects	Related documents
Chaoyang District Government	Phoenix Plan (Fenghuang Jihua) (2009)	*Opinions on Implementing Overseas Talents’ Work in Chaoyang District (2009)*
		*Interim Measures for Chaoyang District to Encourage Overseas High-level Talents to Start-up and Work (2013)*
		*Interim Measures for the Introduction and Subsidy of Excellent Overseas Talents in Chaoyang District (2013)*
Haidian District Government	Haiying Plan (2012)	*Supporting Measures for Promoting Talents’ Innovation and Entrepreneurship Development in Haidian District (2012)*
Beijing’s Economic and Technological Development Zone Government	New Creative Project (Xinchuang Gongcheng) (2015)	n/a
Changping District Government	Changju Plan (2017)	*Interim Measures for Supporting High-level Scientific and Technological Talents in Changping District (2017)*
Tongzhou District Government	Dengta Plan (2017)	n/a
	Yunhe Plan (2017)	

Source: This table was made by the author according to information from the websites of Beijing district governments.

### Appendix 2: Supporting policies for talent introduction plans in Beijing

**Table:**

Year	Documents	Content
2011	*Some opinions on the construction of special talent zone in Zhongguancun National Independent Innovation Demonstration Zone*^a^	‘13 special policies’ for high-level talents
2015	*Several Measures for Deepening the Reform of Talent Management in Zhongguancun*^b^	‘8 measures’ for talents administration
2015–2016	*Notice on Policies and Measures to Support Beijing's Innovative Development*^c^	‘20 measures’ related to exit & entry and residence of foreign talents in Beijing
2018	*Several Measures on Deepening the Reform of Talents Management in Zhongguancun and Constructing a Talents Introduction and Utilization Mechanism with International Competitiveness*^d^	‘New 20 measures’

Source: This table was made by the author according to information from government websites and PKULAW

^a^It was issued on the 4th of March by the Organizational Department of the CCCPC, the National Development and Reform Commission and the Ministry of Education, etc. and 17 government departments in 2011

^b^It was issued on the 21st of October, 2015 by the Organizational Department of the CCCPC, the National Development and Reform Commission and the Ministry of Education, etc. with the issue number [2015] No. 15

^c^It was issued by the Ministry of Public Security with issue number [2015] No. 4076. It was implemented from 2016 in Beijing

^d^It was released in February 2018 by the Central Organization Department, the Ministry of Science and Technology, the Ministry of Public Security, the Ministry of Human Resources and Social Security, the Chinese Association for Science and Technology and five other relevant central and state departments, as well as the Beijing Municipal Committee and Municipal Government

### Appendix 3: Policy documents related to the establishment of Zhongguancun Science Park

**Table:**

Year	Policy documents	Policy outcome
1988	*Provisional Regulations of Beijing Municipality Concerning the New Technology Industry Development Zone*^a^	China’s first National Hi-tech Industrial Development Zone
1999	*Approval of Problems Concerning the Construction of Zhongguancun Science and Technology Park*^b^	
2009	*Approval on Agreeing to Support the Construction of National Independent Innovation Demonstration Zone in Zhongguancun Science and Technology Park*^c^	China’s first national independent innovation demonstration zone
2011	*Some opinions on the construction of special talent zone in Zhongguancun National Independent Innovation Demonstration Zone*^d^	China’s first state-level special talent zone
2011	*Outline of Development Planning for Zhongguancun National Independent Innovation Demonstration Zone (2011–2020)*	
2012	*Regulations on Zhongguancun National Independent Innovation Demonstration Zone*^e^	

Source: This table was made by the author according to information from government websites and PKULAW

^a^It was approved by the State Council of China on the 10th of May, 1988 and issued by Beijing Government on the 20th of May, 1988

^b^It was issued on the 5th of June, 1999 by State Council of China with the issue number [1999] No. 45.

^c^It was issued on the 13th of March, 2009 by State Council of China with issue number [2009] No. 28. Available at Zhongguancun Science Park website: http://zgcgw.beijing.gov.cn/m/zwgk49/zcfg45/gj42/654/index.html

^d^It was issued on the 4th of March by the Organizational Department of the CCCPC, the National Development and Reform Commission and the Ministry of Education, etc. and 17 government departments in 2011

^e^It was approved at the 22nd Session of the Standing Committee of the 13th Beijing People's Congress on the 23rd of December, 2010. Available at the Zhongguancun Science Park website: http://zgcgw.beijing.gov.cn/zgc/zwgk/zcfg18/sfq/139624/index.html

## Footnotes

1 See details on China’s central government website. Available at http://www.gov.cn/zhengce/2017-09/27/content_5227992.htm. Accessed 9th of September 2020.

2 It was issued by the State Council of China on the 26th of September, 1983. Retrieved from PKULAW database with the citation code CLI.2.162031.

3 The full name of the ‘ten thousand talents plan’ is ‘National Special Support Plan for High-level Talents’.

4 It was released by the Beijing Municipal Party Committee and Municipal Government with the issue number [2008] No. 28.

5 It was issued in April 2009 with the issue number [2009] No. 11.

6 It was issued by the Beijing Government with the issue number [2014] No. 3.

7 News from the Economic Daily. 606 Foreigners in Beijing got China’s ‘Green Card’. Available via http://www.sohu.com/a/238683713_118392. Accessed 10th May 2021.

8 News from China Scholars Abroad. Talent discussion: The contribution rate of talents to Beijing's economic growth exceeded 50% for the first time, ranking at the national leading level. Available via https://mp.weixin.qq.com/s?src=3&timestamp=1568043093&ver=1&signature=*llk9isQ0eSSWKLiRuyJNuUr7E0jn3wfeRTSdBvGfoPb-zbkrXiKtjSlj4gcP6N8GTmlU7XgY97PL8ZmvX0ExmQNnE*ZeSqpAFQw-G6-lDzPGky4jyN428jT0r5l*EqSmPqer6g6eDTmI5Ly1nSWwNXIBQGIMW*b5q7A4T1rYyc = . Accessed 20th April 2020.

9 Ibid.

10 News from people.cn. The ‘direct train’ for permanent residence of foreign talents will open next month. Available via http://society.people.com.cn/n1/2016/0225/c1008-28148430.html. Accessed 23rd October 2020.

11 News from people.cn. Zhongguancun attracts foreign talents, the first 7 people take the green card by ‘through train’. Available via http://bj.people.com.cn/n2/2016/0521/c82840-28375886.html. Accessed 15th May 2020.

12 News from the Economic Daily. 606 Foreigners in Beijing got China’s ‘Green Card’. Available via http://www.sohu.com/a/238683713_118392. Accessed 10th May 2021.

13 The first batch of seven foreign high-level talents in Zhongguancun won China's ‘green card’. Available via http://news.takungpao.com/mainland/focus/2016-05/3324041.html. Accessed 21st June 2021.

14 News from People.cn. The history chooses Deng Xiaoping. Available via http://cpc.people.com.cn/n1/2018/0731/c69113-30179947.html. Accessed 30th June 2020.

15 This document is *Report Outline of the State Science and Technology Commission on the Development Guideline for Science and Technology in China*. It was released on the 23rd of October, 1981 by the State Science and Technology Commission of China.

16 *Decision of the CCCPC on the Reform of the Scientific and Technological System* was issued on the 13th of March, 1985 by the CCCPC with the issue number [1985] No. 6. *Decision of the CCCPC on the Reform of the Educational System* was issued on the 27th of May, 1985.

17 Zhao Ziyang's report at the 13th National Congress of the CPC. Available at Chinese Central government portal website, available via http://www.gov.cn/test/2008-07/01/content_1032279.htm. Accessed 16th September 2020.

18 See Outline of Development Planning for Zhongguancun National Independent Innovation Demonstration Zone (2011–2020) and Regulations on Zhongguancun National Independent Innovation Demonstration Zone.

19 It was approved by the Third Plenary Session of the Eighteenth CCCPC on the 12th of November, 2013.

20 More information can be found in the website of the Zhongguancun the Belt and Road Industrial Promotion Association: http://www.zbra.org.cn/index.do#/home.

21 News from Rednet. Speech by vice governor of Zhejiang Province at the ceremony of granting foreigners permanent residence qualification in China by Hunan Provincial Public Security Department. Available via http://www.chinaqw.com/node2/node116/node1165/node1177/node1212/node1215/userobject6ai66417.html. Accessed 25th May 2021.

22 News from the time weekly. Experts suggest reducing China's green card application threshold and introducing foreign advanced assessment system. Available via https://pkulaw.cn/fulltext_form.aspx?Db=news&Gid=3fe97b12f3b6ad60bdfb&keyword=&EncodingName=&Search_Mode=&Search_IsTitle=0. Accessed 30th June 2021.

23 This was a figure released by Lin Xu, then the Member of the CPPCC National Committee, Director-General of the National Han Office and Director-General of the Headquarters of Confucius Institute in March 2014.

24 News from Chinaqw. Chinese media are concerned about China's lowering the threshold for introducing overseas talents, and it is no longer difficult to obtain a green card. Available via http://www.chinaqw.com/jjkj/2015/01-27/35448.shtml. Accessed 30th June 2020.

25 Namely the permanent residence card.

26 News from Chinaqw. How to get the ‘most difficult’ green card in China. Available via http://www.gqb.gov.cn/news/2015/1203/37276.shtml. Accessed 30th June 2020.

27 News from Chinanews.com. Beijing official explanation of 'permanent residence points system for foreign talents in China’. Available via https://www.chinanews.com.cn/hr/2017/01-15/8124946.shtml. Accessed 4th July 2021.
